# Rapamycin Augments the NMDA-Mediated TNF Suppression of MRSA-Stimulated RAW264.7 Murine Macrophages

**DOI:** 10.1155/2012/542727

**Published:** 2012-10-10

**Authors:** Thomas Spentzas, Rebekah K. H. Shappley, Fabio Savorgnan, Elizabeth Meals, B. Keith English

**Affiliations:** ^1^Division of Pediatric Critical Care, Department of Pediatrics, University of Tennessee Health Science Center, Memphis, TN 38103, USA; ^2^Le Bonheur Children's Hospital, 50 N. Dunlap, Memphis, TN 38103, USA; ^3^Children's Foundation Research Center, Memphis, TN 38103, USA; ^4^Department of Pediatrics, University of Tennessee Health Science Center, Memphis, TN 38103, USA; ^5^Division of Pediatric Infectious Diseases, Department of Pediatrics, University of Tennessee Health Science Center, Memphis, TN 38103, USA

## Abstract

*Background*. Methicillin-resistant *Staphylococcus aureus* (MRSA) can stimulate massive cytokine release. Ketamine suppresses tumor necrosis factor (TNF) secretion by MRSA-stimulated RAW264.7 macrophages, and the mechanism likely involves N-methyl-D-aspartic acid (NMDA) receptor antagonism. The downstream effects of NMDA-mediated TNF suppression, specifically the PI3K/Akt and mTOR modulation, have not been described. *Methods*. RAW264.7 cells were stimulated for 18 hrs with 10^5^ to 10^7^ CFU/mL inocula of either of two prototypical community-acquired- (CA-) MRSA isolates, USA300 strain LAC and USA400 strain MW2. Then we added the NMDA inhibitors ketamine or 2R-amino-5-phosphonopentanoate (AP5), NMDA substrate, LY294002, and rapamycin in various combinations. *Results*. NMDA inhibition suppressed TNF secretion by almost a third compared to the no-ketamine control. When NMDA substrate was added, the TNF secretion increased by 10%. Addition of LY294002 suppressed TNF production by macrophages by 20%. Rapamycin exhibited a concentration-dependent TNF induction-suppression response: induction at doses of 0.1 and 1 ng/mL and suppression at 10 and 100 ng/mL. Induction of TNF was abolished when LY294002 was added and the suppression became uniform. Ketamine-induced suppression of TNF secretion was intensified 10–15% when rapamycin was added, but not when LY294002 was added. *Conclusion*. These findings suggest that NMDA-induced TNF suppression can be augmented by concurrent mTOR inhibition.

## 1. Introduction

Sepsis is a major cause of morbidity and mortality, with an estimated incidence in the pediatric population of 0.56 cases per 1000 annually. Staphylococcal infections are the leading cause of sepsis [1, 2]. Bacterial components and secreted toxins can stimulate massive cytokine release and lead to septic shock, acute respiratory distress syndrome, and death [3]. It is likely that strategies designed to modulate the excessive and prolonged host inflammatory response could improve the outcome of such infections. Macrophage cells are the main source of proinflammatory cytokines, including interleukins such as IL-1*β*, IL-6, IL-8, IL-12, and tumor necrosis factor (TNF) secreted in response to bacterial stimulation [4, 5]. TNF is a multifunctional cytokine that is produced primarily by activated macrophages and plays an important role in endotoxic shock, immunity, inflammation, antiviral responses, and diseases such as cachexia and non-insulin-dependent diabetes [6–8]. In our previous studies we showed that secretion of TNF depends on the expression of certain virulent factors, specifically four cytolytic phenol-soluble modulin (PSM) peptides [9]. The inflammatory response can be decreased in the presence of a nonlytic antibiotic, daptomycin [9, 10]. We also showed that one of most recommended anesthetics for septic shock, ketamine, can suppress the TNF secretion mechanism independently of PSM expression or nonlytic antibiotic action [11]. Tumor necrosis factor activates the nuclear factor kappa-light chain enhancer of activated B cells (NF-*κ*B), which is normally inactive and sequestered in the cytoplasm combined with an inhibitor [12]. The activation of transcription factor NF-*κ*B by TNF is mediated via the phosphatidylinositol 3-kinase (PI3 K) pathway [12].

Ketamine's action begins with antagonism of the N-methyl-D-aspartate (NMDA) receptor. Its effects on the downstream pathway are not known. It has been shown recently that ketamine has a rapid antidepressant effect that is attributed to activation of the mammalian target of rapamycin (mTOR) [13]. Calcineurin inhibitors FK506 and cyclosporine can suppress the NMDA receptors in the hippocampus [14]. Rapamycin, or sirolimus, is an mTOR inhibitor with a macrolide antibiotic structure, useful in prevention of allograft loss [15–21]. It was first discovered in the soil of Easter Island (Rapa Nui) and is a product of *Streptomyces hygroscopicus* [15, 22]. It exerts its immunosuppressive effect in the same way as cyclosporine A and FK506, by binding to immunophilins and interfering with the signaling pathways of T-lymphocyte proliferation [23]. Rapamycin binds to the cytosolic protein FK-binding protein 12 (FKBP12) in the same way as the structurally similar FK506 [24]. The FKBP12 protein is regulated by the human FRAP1 gene located on chromosome one and is a phosphatidyl-inositol kinase (stress mediator) [25, 26]. The rapamycin-FKBP12 complex binds to the mTOR complex 1 (mTORC1) [27–29], which is a serine/threonine phosphokinase and functions as a redox sensor, regulating cell growth, proliferation, motility, and survival, as well as protein synthesis and transcription [30]. Recent studies show suppression of TNF production by vascular smooth muscle cells stimulated by lipopolysaccharide (LPS) when rapamycin was added at 1 ng/mL [31]. Rapamycin induces apoptosis in monocyte- and CD34-derived dendritic cells, but not in monocytes or macrophages [32] Some studies in human endothelial cells showed that the addition of rapamycin in doses close to 10 and 100 ng/mL might cause accelerated cell death and loss of cell mobility [33]. The present study further explores the NMDA anti-inflammatory pathway and, specifically, the role of the PI3K/Akt system, mTOR, and rapamycin in the community-associated- (CA-) methicillin-resistant *Staphylococcus aureus*- (MRSA-) stimulated RAW264.7 murine macrophage model. It assesses whether the NMDA-induced TNF inhibition can be augmented with rapamycin, according to the observed ketamine rapamycin antidepression model [13]. The combination of an NMDA and mTOR inhibitory effect on the TNF suppressive response can be an important clinical approach in sepsis.

## 2. Materials and Methods

### 2.1. Bacteria

For these studies, we utilized two well-characterized clinical isolates, LAC (Los Angeles County), representative of the USA300 group of organisms, closely related to the dominant CA-MRSA clone associated with soft tissue infections and serious invasive disease in the Memphis area and MW2, another clinical isolate from a Midwestern child with fatal CA-MRSA sepsis, representative of the USA400 group of organisms that constitute the other main lineage of CA-MRSA isolates in the United States [34, 35].

Otto and DeLeo and colleagues recently generated isogenic strains of LAC and MW2 engineered to lack expression of PSMs or *δ*-toxin and generously provided these strains for our studies: in this experiment we used the Δ-PSM-*α*-LAC-MRSA, a stable strain with a well-described diminished TNF response in comparison to the isogenic wild-type strain [11, 36].

Bacteria were grown to late logarithmic phase at 37°C in tryptic soy broth (Becton, Dickinson and Company, Sparks, MD, USA) and washed three times in endotoxin-free phosphate-buffered saline. Concentrations were determined by colony counts. A range of concentrations of bacteria (10^5^–10^7^ CFU/mL) was studied, based upon our previously published data with other CA-MRSA strains and our preliminary experiments [9–11]. The minimum inhibitory concentrations (MICs) for these strains were determined by using the *E*-test method in the microbiology laboratory at Le Bonheur Children's Hospital (LBCH). Both strains were fully susceptible to vancomycin (LAC: MIC vancomycin 1.0 *μ*g/mL; MW2: MIC vancomycin < 0.5 *μ*g/mL). Beside bacteria for macrophage stimulation, we used bacterial products such as lipopolysaccharide (LPS), a component of Gram-negative bacteria cell walls, and lipoteichoic acid (LTA), a component of Gram-positive bacteria cell walls, in the experiments with rapamycin. This was done to confirm that the rapamycin effects are independent of any rapamycin-MRSA interaction. 

### 2.2. Cell Culture

RAW264.7 murine macrophage-like cells were purchased from the ATCC and cultured in Dulbecco's modified Eagle's medium (Mediatech Inc., Herndon, VA, USA) supplemented with 10% fetal bovine serum (HyClone, Logan, UT, USA) and 2 mM glutamine (GIBCO, Grand Island, NY, USA). Experiments were done in 24-well tissue culture plates (Becton Dickinson, Franklin Lakes, NJ, USA) targeting 1 × 10^6^ cells per well. 

Vancomycin, at a clinically achievable concentration of 20 *μ*g/mL, was added to the cell cultures immediately before the addition of live staphylococci (10^5^–10^7^ CFU/mL). Cells were then incubated for 18 hours [10]. Vancomycin was purchased via the Department of Pharmacy at LBCH from Hospira (Lake Forest, IL, USA). It suppresses the uncontrolled growth of MRSA and massive TNF production.

These experiments were repeated in parallel and in various combinations in the presence of ketamine (100 *μ*M), AP5 (3 mM), LY-294002 (500 nM), and NMDA substrate (30 *μ*M). Rapamycin was added at concentrations of 0.1, 1, 10, and 100 ng/mL: the relevance of the concentrations selected have been described previously [32, 33]. The modulation of MRSA-stimulated macrophage TNF production by ketamine was tested at concentrations of 10, 50, 100, and 150 *μ*M, and the selected concentration (100 *μ*M) is based on the achievable anesthetic concentrations, what is reported in the existing literature and our own work [5, 11, 37–43]. The source of ketamine was Ketalar, a racemic mixture (1 : 1) of optically active isomers (R and L) of this drug, purchased from the LBCH pharmacy. Emphasis in the experiment was placed on correlation with the clinical situation; thus, racemic ketamine, the most common clinically used product, was selected. AP5, NMDA, and rapamycin were purchased from Sigma Chemical Co. (St. Louis, MO, USA).

After incubation, cell-free supernatants were collected and assayed for TNF concentrations by using a solid-phase sandwich enzyme-linked immunoabsorbent assay as specified by the manufacturer (eBioscience, San Diego, CA, USA). TNF is a key cytokine produced by macrophages during MRSA stimulation. We measured secretion of other cytokines and found that IL-6 and IL-12 secretions were strongly correlated with TNF secretion in response to these bacteria (*r* ^2^ = 0.92 and 0.91, resp.). These findings were consistent with our previous studies [9, 11]. We focused on TNF secretion for these studies.

The tested concentrations of vancomycin, ketamine, AP5, NMDA, LY294002, and rapamycin had no effect on the viability of the RAW264.7 cells, as determined by visual inspection of the monolayer, low-power microscopic inspection of the monolayer, and exclusion of 0.2% trypan blue dye. Cell viability was confirmed using 3-(4,5-dimethylthiazol-2-yl)-5-(3-carboxymethoxyphenyl)-2-(4-sulfophenyl)-2H-tetrazolium, inner salt (MTS) according to the manufacturer's instructions (Promega). The MTS reagent is reduced by metabolically active cells into a colored formazan product whose absorbance is then measured. In brief, MTS solution was added to wells of a 96-well microtiter plate, and the cells were incubated for 2 h. The absorbance at 490 nm was then measured.

For the comparison experiments, TNF secretion measurements were validated with an average of at least three well replicates and each of the experiments was repeated at least three times (a total of nine samples). The experiments were performed separately for LAC wild-type, Δ-PSM-*α* LAC, and for MW2 wild-type MRSA strains. We confirmed uniform MRSA strain response in all of the experiments. There is an intrinsic experimental variation of absolute maximum values of TNF production in different cell culture flasks due to the unique cell culture and endogenous macrophage differences, a finding consistent in all our previous studies [9–11]. To minimize the variation, we conducted each experiment with cells from the same population. We standardized the responses (TNF production) to experimentally set “control” responses, that is, stimulation without medication exposure or other modifications. We reported the results as percent of the control. The percent expression helps to understand better the treatment effects. From our previous experience with the macrophage sepsis model as well as from clinical observation, an “ideal response” is a consistent TNF reduction 10–40%. Reduced TNF levels (but not totally suppressed) are associated with best clinical outcome [44–48]. The percentile can easily be transformed to the actual value because the actual control value (pg/mL) for each experiment is given; for example, when the response is 80% of the control and the actual control value is 33,561 pg/mL, then the response is 0.8 × 33,561 pg/mL = 26,849 pg/mL. Figures 4(a) and 4(b) depict the same results of Figure 4(a) as fold change of the control and of Figure 4(b) as pg/mL TNF production. 

### 2.3. Data Analysis

The results were analyzed with R 2.13.1 and the ggplot2 graph package. They were validated by SPSS19-IBM. The experimental design consisted of factorial multiple measurements. The normality was assessed with the Kolmogorov-Smirnov test and a plot. The ANOVAs are between independent groups, and we tested the homogeneity of the variance with Levene's test. We set preplanned (*a priori*) contrasts; that is, we set all our comparisons in advance of multiple setting experiments. Significant differences were presumed at a probability value of *P* < 0.05. When post hoc tests were used we applied the Bonferroni correction. The results were graphed as fold of increase compared to the control using error bars with 95% confidence intervals.

## 3. Results

NMDA pathway inhibition or induction modulates the TNF response of RAW264.7 murine macrophages stimulated by CA-MRSA wild-type strains MW2 and LAC.

Our goal was to test the NMDA pathway downstream inhibition (PI3 K) or NMDA induction (NMDA substrate) alone or concurrently with NMDA inhibition (ketamine and AP5). RAW264.7 murine macrophages were stimulated for 18 hrs with 10^6^–10^7^ CFU/mL of MW2 wild-type and LAC wild-type CA-MRSA. Bacterially stimulated macrophage TNF secretion without exposure to ketamine, AP5 (APV), LY-294002, or NMDA substrate served as the control. The control TNF production was contrasted with stimulation in the presence of the PI3 K inhibitor LY294002 (500 nM), NMDA substrate (30 *μ*M), and a combination of both NMDA substrate and LY294002. The same sequence of exposure (LY294002, NMDA substrate, a combination of LY294002 and NMDA substrate) was repeated in the presence of NMDA inhibitors ketamine (100 *μ*M) or AP5 (3 mM). 

**Figure 1:**
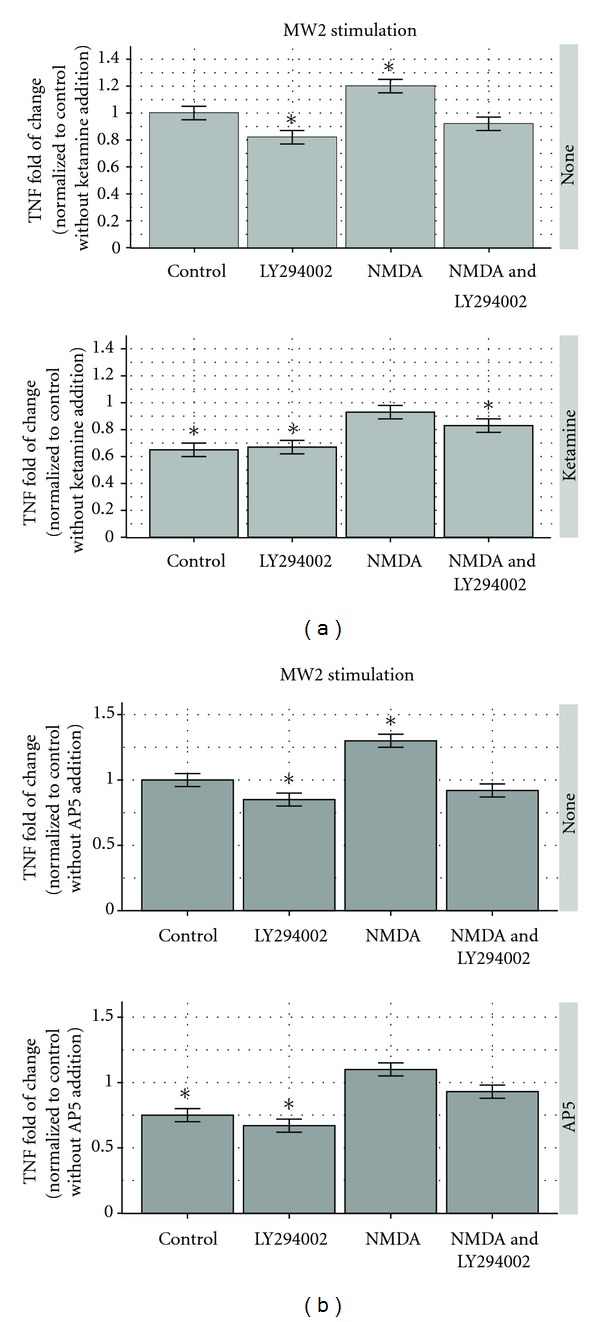
(a) Ketamine and the PI3 K/Akt inhibitor LY294002 modify TNF production by RAW264.7 murine macrophages. In the upper panel, compared to the not treated control-first column, MRSA-MW2 (USA400) stimulated less TNF secretion (18%) by macrophages when exposed to LY294002 (second column), more secretion (20%) when exposed to NMDA substrate (third column), and was not significantly different from the control TNF secretion (8% decrease) when both NMDA and LY294002 were present (fourth column). In the lower panel, ketamine (first column) was added and suppressed TNF production 35% less than the control (upper panel, first column). Ketamine and LY294002 (lower panel, second column) also caused TNF suppression (33%), but when compared with ketamine alone (lower panel, first column), the suppression was not significantly different. Ketamine and NMDA caused a 7% TNF increase (not different from the control at *P* < 0.05), and ketamine-NMDA with LY294002 caused 17% TNF suppression. The results are depicted as fold of change compared to the control with 95% confidence intervals shown as “error bars.” *P < 0.05. The control response was 33, 561 ± 842 pg/mL.  (b) AP5 (APV), another NMDA inhibitor, modified TNF production by RAW264.7 murine macrophages in a way similar to that of ketamine. In the upper panel MW2 (USA400) stimulated 15% less TNF secretion by macrophages when exposed to LY294002 (second column), 30% more when exposed to NMDA substrate (third column)—both significantly different from the untreated control (upper panel, first column) at *P* < 0.05. The TNF secretion in the presence of both NMDA and LY294002 (upper panel, fourth column) was not significantly different from the control. In the same experimental sequence in the lower panel, AP5 caused significant suppression of TNF production (25%—lower panel, first column) in a manner similar to that of ketamine (Figure 1, lower panel, first column). Of the rest, only the combination LY294002 and AP5 (second column) caused a significant suppression (33%) compared to control (*P* < 0.05). The results are depicted as fold of change compared to the control with 95% confidence intervals shown as “error bars.” *P < 0.05. The control response was 30,221 ± 604 pg/mL.

The presence of LY294002 suppressed TNF production by 18% (*P* < 0.05). Adding NMDA substrate increased TNF production by 20% (*P* < 0.05). LY294002 in the presence of NMDA substrate decreased TNF secretion by only 8%—a difference not significantly different than the control. The addition of ketamine decreased TNF production by 35% (*P* < 0.05). When ketamine and LY294002 were added together, the reduction in TNF was significantly (33%) different than the control (*P* < 0.05) but not significantly different from the concentration induced by ketamine alone. NMDA and ketamine induced 7% suppression, which was not significantly different from the control response. The ketamine-NMDA-LY294002 response was decreased 17% and was significantly different from the control (*P* < 0.05). The results are depicted in Figure 1(a).

The results were similar when AP5 (APV) was used instead of ketamine in the same sequence of experimental settings (Figure 1(b) depicts the AP5 effect on TNF production after CA-MRSA MW2 stimulation). In this experiment, there was a 15% decrease in TNF secretion in comparison to the control when exposed to LY294002 and a 30% increase when exposed to NMDA substrate—both significantly different at *P* < 0.05. The level of TNF secretion in the presences of a combination of NMDA and LY294002 (8% decrease) was not significantly different from the control. AP5 caused 25% suppression of TNF production and AP5 plus LY294002 caused 33% suppression, both significant differences at *P* < 0.05. AP5 with NMDA and AP5 with NMDA and LY294002 (10% increase and 7% decrease) were not significantly different from the control. The responses were similar for both CA-MRSA strains MW2 and LAC (not shown). 

IL-6 and IL-12 production demonstrated similar patterns of response when substrate NMDA was added—an increase of 13% and 11%, respectively (*P* < 0.05), and suppression with ketamine treatment of 25% and 22% of the control (*P* < 0.05). The control response for IL-6 ± standard error (SE) was 19 ± 0.6 pg/mL, and the response for IL-12 was 127 ± 3.2 pg/mL.

**Effects of Rapamycin on TNF Production by Bacterially Stimulated RAW264.7 Macrophages**

Exposing unstimulated macrophages to rapamycin had no effect. Visual inspection of the monolayer, low-power microscopic inspection, and exclusion of 0.2% trypan blue dye confirmed that at least 90% of macrophages were viable after 18–24 hours of exposure. Thus, rapamycin did not affect the viability of macrophages during the experimental period. Neither the interaction of rapamycin with bacterially stimulated macrophages nor dose responses have been described. These had to be established before we considered manipulations of the NMDA-PI3 K-mTOR pathway. We tested several models of stimulation, including MW2 MRSA (wild-type), LAC MRSA (wild-type), Δ-PSM-*α*-LAC-MRSA (a genetically engineered strain missing the PSM-*α* antigens) [9] as well as bacterial products lipopolysaccharide (LPS), a component of Gram-negative bacteria cell walls, and lipoteichoic acid (LTA), a component of Gram-positive bacteria cell walls. These tests were necessary to assess the uniformity of the rapamycin response.

**Figure 2:**
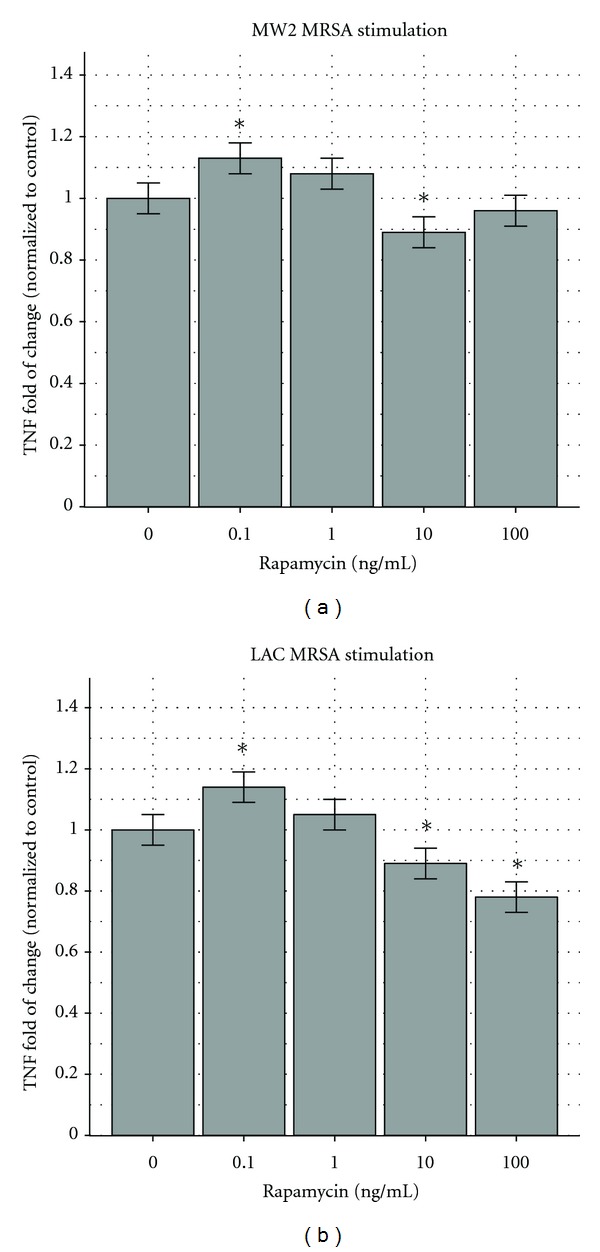
(a) Effects of rapamycin on RAW264.7 murine macrophages stimulated by MW2 MRSA. The MW2 (USA400) MRSA strain stimulated more TNF secretion by RAW264.7 murine macrophages when exposed to rapamycin at concentrations of 0.1 and 1 ng/mL and less when exposed to rapamycin at 10 and 100 ng/mL. The response at 0.1 ng/mL (second column) and 10 ng/mL (fourth column) represented an increase of 13% and a decrease of 11%, both significantly different (*P* < 0.05) from the untreated control (first column). The responses at 1 ng/mL (third column) and 100 ng/mL (fifth column) were an 8% increase and 4% decrease, which were not significantly different from the control. The results are depicted as fold of change compared to the control with 95% confidence intervals shown as “error bars.” *P < 0.05. The control response was 37, 632 ± 941 pg/mL. (b) Effects of rapamycin on RAW264.7 murine macrophages stimulated by LAC MRSA. The LAC (USA300) MRSA strain stimulated more TNF secretion by RAW264.7 murine macrophages when exposed to rapamycin at 0.1 and 1 ng/mL and less when exposed to rapamycin at 10 and 100 ng/mL. The response at 0.1 ng/mL (second column) had an increase of 14%, significantly different (*P* < 0.05) from the untreated control (first column). The response at 1 ng/mL (third column) presented an increase of 9%, which did not reach statistical significance. The responses at 10 ng/mL (fourth column) and 100 ng/mL (fifth column) demonstrated decreases of 11% and 22%—significantly different from the control at *P* < 0.05. The results are depicted as fold of change compared to the control with 95% confidence intervals shown as “error bars.” *P < 0.05. The control response was 27,965 pg/mL ± 699 pg/mL.

Macrophages stimulated by MW2 (wild-type CA-MRSA) in the presence of 0.1 and 1 ng/mL of rapamycin exhibited an increase in production of TNF of 13% and 8%, respectively, but in the presence of 10 and 100 ng/mL they exhibited a decrease in TNF production (11% and 4%, resp.); the results are depicted in Figure 2(a). The same biphasic concentration-dependent response was seen with LAC wild-type MRSA: 14% and 5% increase with 0.1 and 1 ng/mL of rapamycin, respectively, and 11% and 22% decrease with 10 and 100 ng/mL rapamycin (Figure 2(b)). 

**Figure 3:**
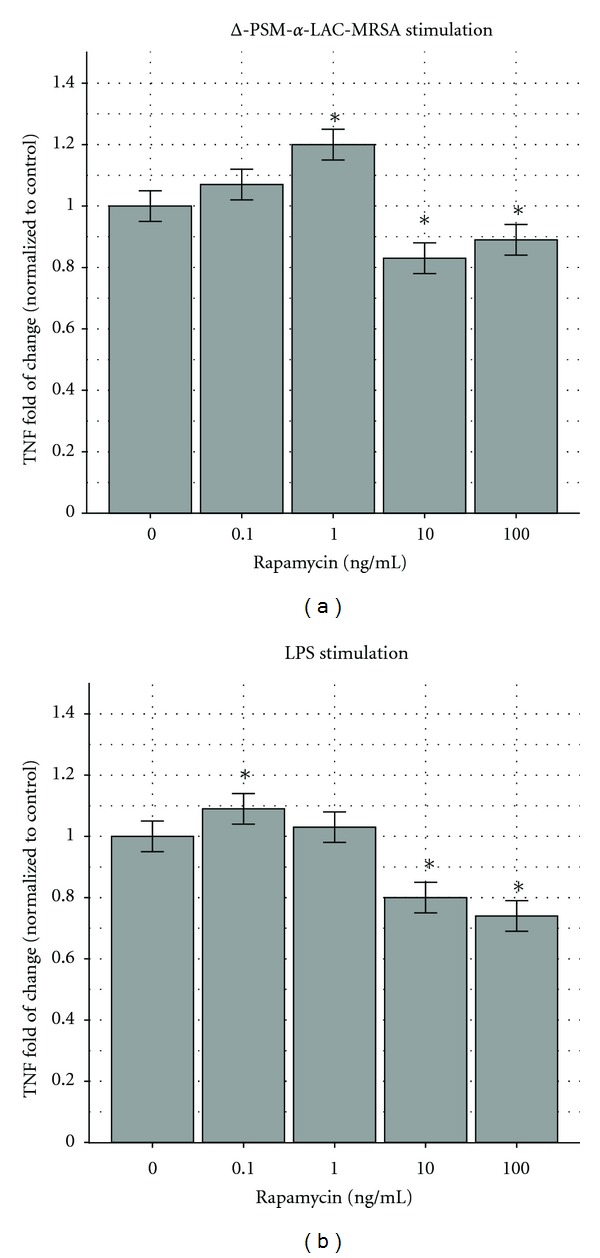
(a) Effects of rapamycin on MRSA RAW264.7 murine macrophages stimulated by Δ-PSM-*α* LAC. Δ-PSM-*α* LAC (USA300 PSM-*α*-depleted strain) stimulated more TNF secretion by RAW264.7 murine macrophages when exposed to rapamycin at 0.1 and 1 ng/mL and less when exposed to rapamycin at 10 and 100 ng/mL. The response at 0.1 ng/mL (first column) had an increase of 7%, not significantly different (*P* < 0.05) than the untreated control (first column). The response at 1 ng/mL (third column) had an increase of 20%, and the responses at 10 ng/mL (fourth column) and 100 ng/mL (fifth column) demonstrated decreases of 17% and 11%, all significantly different from the control (*P* < 0.05). The results are depicted as fold of change compared to the control with 95% confidence intervals shown as “error bars.” *P < 0.05. The control response was 28, 554 ± 571 pg/mL. (b) Effects of rapamycin on RAW264.7 murine macrophages stimulated by LPS. Lipopolysaccharide (LPS) stimulated more TNF secretion by RAW264.7 murine macrophages when exposed to rapamycin at 0.1 and 1 ng/mL and less when exposed to concentrations of 10 and 100 ng/mL. The response at 0.1 ng/mL had an increase of 9%, significantly different (*P* < 0.05) from the untreated control (first column). The response at 1 ng/mL had an increase of 7% (not significantly different), and the responses at 10 ng/mL (fourth column) and 100 ng/mL (fifth column) demonstrated decreases of 20% and 26%, both significantly different from the control (*P* < 0.05). The results are depicted as fold of change compared to the control with 95% confidence intervals shown as “error bars.” *P < 0.05. The control response was 32,486 pg/mL ± 650 pg/mL.

Δ-PSM-*α*-LAC-MRSA stimulation also caused a biphasic response: 7% and 20% increase with 0.1 and 1 ng/mL of rapamycin, and 17% and 11% decrease with 10 and 100 ng/mL of rapamycin (Figure 3(a)). We also tested macrophages stimulated with LPS in the presence of 0.1, 1, 10 and 100 ng/mL of rapamycin, and the results were increases of 9% and 3% and decreases of 20% and 26%, respectively (Figure 3(b)). 

**Figure 4:**
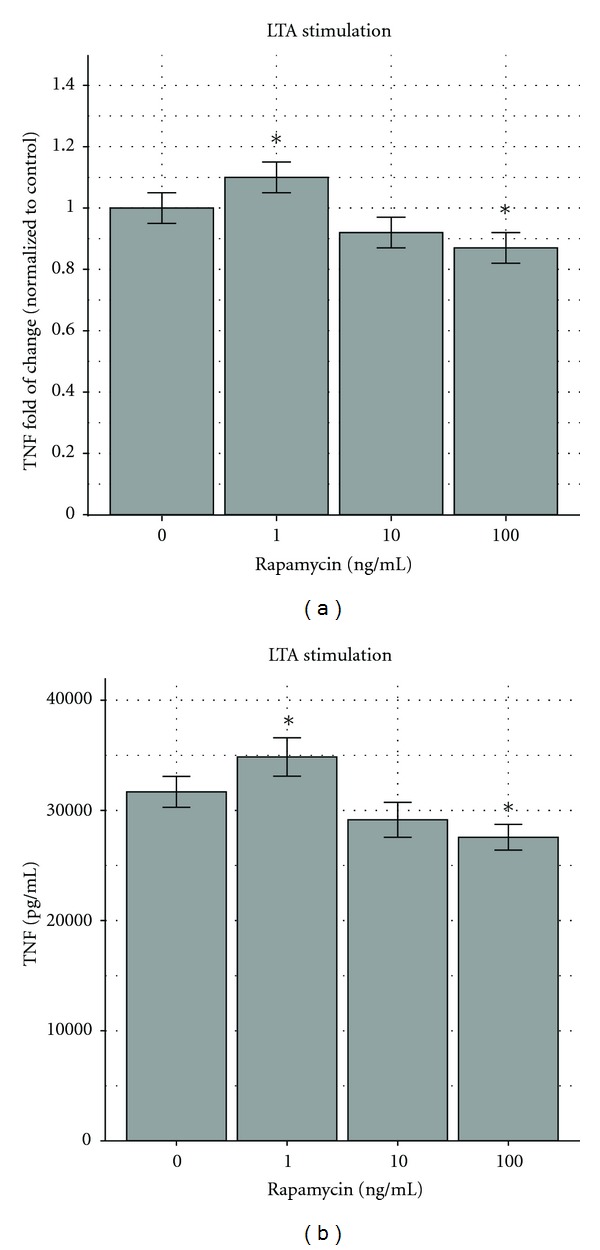
(a) Effects of rapamycin on RAW264.7 murine macrophages stimulated by LTA. Lipoteichoic acid (LTA) stimulated more TNF secretion by RAW264.7 murine macrophages when exposed to rapamycin at 0.1 and 1 ng/mL and less when exposed to rapamycin at 100 ng/mL. The response at 0.1 ng/mL had an increase of 10%, significantly different (*P* < 0.05) from the untreated control (first column). The response at 10 ng/mL (third column) had a decrease of 8% (not significantly different), and the response at 100 ng/mL (fourth column) demonstrated a 13% decrease, significantly different from the control (*P* < 0.05). The results are depicted as fold of change compared to the control with 95% confidence intervals shown as “error bars.” *P < 0.05. The control response was 31,684 pg/mL ± 713 pg/mL. (b) Effects of rapamycin on RAW264.7 murine macrophages stimulated by LTA. Lipoteichoic acid (LTA) stimulated more TNF secretion by RAW264.7 murine macrophages when exposed to rapamycin at 0.1 and 1 ng/mL and less when exposed to rapamycin at 100 ng/mL. (b) depicts (a) results with actual TNF (pg/mL) secretion, that is, 31.684(713), 34.852(889), 29149(809), and 27.565(593) pg/mL, respectively.

Macrophages stimulated with LTA showed a 10% increase in the presence of 1 ng/mL of rapamycin, and a decrease of 8% and 13% with 10 and 100 ng/mL, respectively (Figure 4(a)). Thus, our data indicate that rapamycin modifies the secretion of TNF from bacterially stimulated macrophages in a dose-dependent biphasic way.

The analysis of specimens using IL-6 and IL-12 in the MRSA-MW2 stimulation model revealed suppression at the 10 ng/mL and 100 ng/mL rapamycin doses: 10% and 11% for IL-6 and 11% and 13% for IL-12, respectively (*P* < 0.05 for both). The responses in the presence of 0.1 and 1 ng/mL rapamycin for both IL-6 and IL-12 were not significantly different from the control. Suppression was noticed also with MRSA-LAC stimulation at 100 ng/mL rapamycin (IL-6, 9%; IL-12, 11%; *P* < 0.05). In these experiments, the control response for IL-6 was 21 ± 0.8 pg/mL and the control response for IL-12 was 131 pg/mL ± 4.1 pg/mL.

Rapamycin increases ketamine suppression of TNF production by bacterially stimulated RAW264.7 macrophages. LY294002 does not cause an additional effect but makes the response independent of the dose.

**Figure 5:**
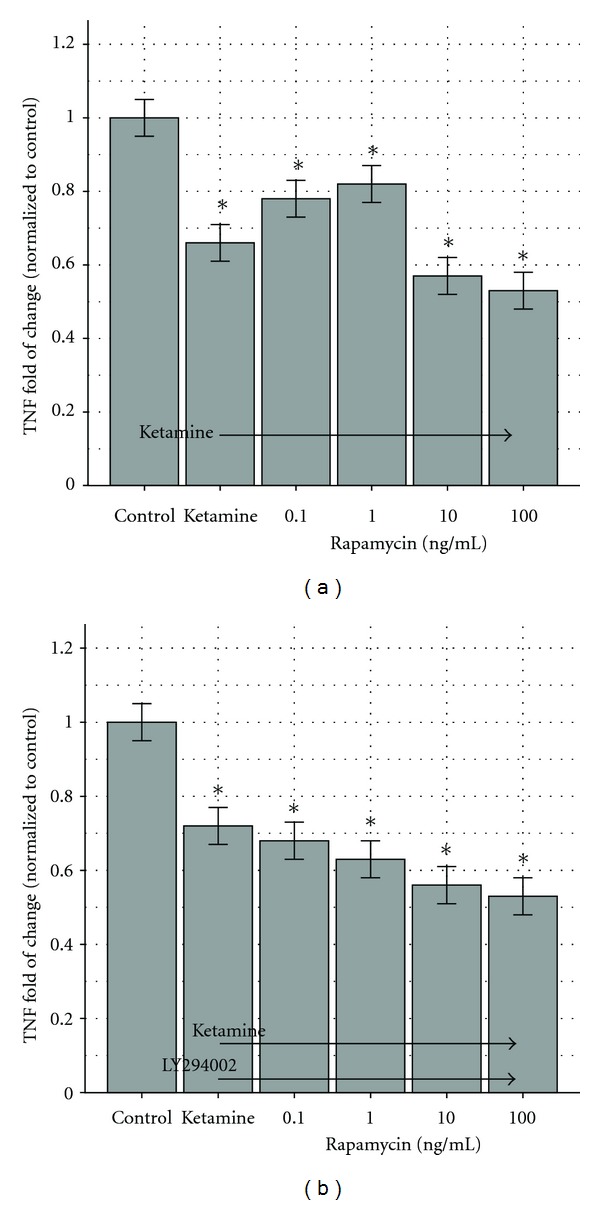
(a) Ketamine and rapamycin modify TNF production by RAW264.7 murine macrophages. LAC wild-type MRSA (USA300) stimulated 34% less TNF secretion by macrophages when exposed to ketamine (second column), and 43% and 47% less than the control (first column) when exposed to a combination of ketamine and rapamycin at 10 ng/mL (fifth column) and 100 ng/mL (sixth column). When ketamine was combined with rapamycin at 0.1 ng/mL (third column) and 1 ng/mL (fourth column), TNF production was suppressed to a lesser extent (22% and 18%, resp.). The suppression observed at 0.1 and 1 ng/mL is different at *P* < 0.05 than the one observed at 10 and 100 ng/mL, and all concentrations decreased TNF production compared to the control (*P* < 0.05). The control (first column) is macrophage stimulation without ketamine or rapamycin. The results are depicted as fold of change compared to the control with 95% confidence intervals shown as “error bars.” *P < 0.05. The control response was 29, 229 ± 731 pg/mL. (b) Ketamine, rapamycin, and LY294002 modify TNF production by RAW264.7 murine macrophages. The LAC wild-type MRSA (USA300) strain stimulated less TNF secretion by macrophages when exposed to ketamine, LY294002, and rapamycin. The presence of LY294002 abolished the dose response depicted in Figure 8. Specifically, the reduction with ketamine and LY294002 was 28% (second column). Ketamine plus LY294002 and rapamycin at 0.1 ng/mL (third column) and 1 ng/mL (fourth column) caused reductions of 32% and 37%, respectively. With rapamycin doses of 10 ng/mL (fifth column) and 100 ng/mL (sixth column), the reductions were 44% and 47%. Ketamine with low doses (0.1 and 1 ng/mL) of rapamycin did not give results significantly different from ketamine alone, but higher doses (10 and 100 ng/mL) caused augmented TNF suppression (*P* < 0.05). The control was macrophage stimulation without ketamine, rapamycin, or LY294002, and the second column shows RAW264.7 cell stimulation in the presence of ketamine only. The results are depicted as fold of change compared to the control with 95% confidence intervals shown as “error bars.” *P < 0.05. The control response was 28, 445 ± 701 pg/mL.

In these sets of experiments, the macrophages stimulated by LAC wild-type CA-MRSA were exposed to modulating combinations of ketamine and rapamycin. The control was TNF produced from stimulated cells without any exposure to modulators. Ketamine without rapamycin suppressed TNF production by 34% (*P* < 0.05). When ketamine was combined with rapamycin at 0.1 and 1 ng/mL, we noticed a decrease in TNF secretion of 22% and 18%, respectively, compared to the untreated control (*P* < 0.05; Figure 5(a)). Thus, TNF production was suppressed less at low concentrations of rapamycin in comparison to ketamine alone.

In the presence of ketamine and rapamycin at 10 and 100 ng/mL, we noticed a decrease of TNF production of 43% and 47% (*P* < 0.05) in comparison to the control—a stronger suppression than that achieved with ketamine alone. 

Subsequently, we repeated the exposure in the presence of LY294002. TNF suppression became more uniform, that is, 28% for ketamine alone (*P* < 0.05), 32% for ketamine + LY294002 + rapamycin at 0.1 ng/mL (*P* < 0.05), 37% for ketamine + LY294002 + rapamycin at 1 ng/mL (*P* < 0.05), 44% for ketamine + LY294002 + rapamycin at 10 ng/mL (*P* < 0.05), and 47% for ketamine + LY294002 + rapamycin at 100 ng/mL (*P* < 0.05). The results are depicted in Figure 5(b). 

## 4. Discussion

Ketamine suppressed TNF secretion by murine macrophages stimulated with CA-MRSA bacteria in the presence of antibiotics by almost a third of the control value. AP5, another NMDA inhibitor, showed a similar pattern of suppression. When NMDA substrate was added to the CA-MRSA-stimulated macrophages, we observed a 10% increase in TNF production in relation to the control, noticeable even in the presence of suppressors of ketamine or AP5. These findings are suggestive of an anti-inflammatory NMDA-mediated mechanism.

The ketamine-induced suppression was intensified when high doses (10 and 100 ng/mL) of rapamycin were added, but not with LY294002. LY294002 is a PI3 K inhibitor, and although it did not exhibit an additive effect with ketamine, it suppressed TNF production in the CA-MRSA macrophage stimulation model by 20%. We attribute this finding to a common pathway inhibition (PI3 K) of ketamine and LY294002, which of course cannot be augmented by synergy.

Rapamycin exhibited a concentration-dependent TNF induction-suppression response when added to bacterially stimulated RAW264.7 cells. There is approximately a 10% induction at lower concentrations of rapamycin (0.1 and 1 ng/mL) and approximately 10% suppression at higher concentrations (10 and 100 ng/mL). This biphasic response to rapamycin was observed after stimulation by MRSA-MW2 wild type and LAC wild type, Δ-PSM-*α*-LAC, LPS, or LTA. Thus, there is an intrinsic system response to rapamycin. Adding ketamine (100 *μ*M) to the CA-MRSA monocyte stimulation model pretreated with the concentration gradient of rapamycin caused 20% TNF suppression at 0.1 and 1 ng/mL of rapamycin and 40–50% TNF suppression at 10 and 100 ng/mL. The concentration-based biphasic response was abolished when the PI3 K/Akt inhibitor LY294002 was added and suppression became uniform (see difference between Figures 5(a) and 5(b)). The concentration-dependent response to rapamycin may be attributed to different actions of different mTOR complexes, that is, mTORC1 and mTORC2. Through the eukaryotic translation factor 4E, mTORC1 binds protein 1 (4EBP1) and ribosomal S6 kinase (S6 K1) and inhibits PI3 K/Akt. Rapamycin FKBP 12 binds directly to mTORC1 and suppresses 4EBP1 and S6KI [49, 50]. Thus, rapamycin can cause the PI3 K/Akt pathway to run uninhibited and possibly increase TNF at lower concentrations, as we observed.

mTORC2 is an inductive part of PI3 K/Akt. In the past, the rapamycin FKBP 12 complex was considered to be unable to modify mTORC2, but recent evidence shows the interaction to be time, cell, and dose dependent [51, 52]. Possibly at higher concentrations rapamycin can suppress PI3 K/Akt and therefore suppressed TNF production. Thus, the response differences shown in Figures 5(a) and 5(b) may be attributed to an interplay of concentration-dependent mTORC1 and mTORC2 inhibition augmented by LY294002 action on PI3 K/Akt. More work needs to be done to clarify these differences.

Our data suggest that a very desirable moderation of the TNF produced by CA-MRSA-stimulated RAW264.7 macrophages can be achieved with a combination of NMDA and mTOR inhibition, possibly with “higher” rapamycin doses, although more work is necessary to help us understand these relationships.

## 5. Conclusions

NMDA inhibitors mediate an anti-inflammatory action on bacterially stimulated macrophages. The anti-inflammatory response can be augmented by treatment of cells with rapamycin at doses of 10 and 100 ng/mL. Such inhibition may be related to activation of the mTOR1/mTOR2 pathway. Such findings may have a significant impact on the immunological treatment of sepsis.

## References

[1] Moran GJ, Krishnadasan A, Gorwitz RJ (2006). Methicillin-resistant *S. aureus* infections among patients in the emergency department. *The New England Journal of Medicine*.

[2] Watson RS, Carcillo JA, Linde-Zwirble WT, Clermont G, Lidicker J, Angus DC (2003). The epidemiology of severe sepsis in children in the United States. *American Journal of Respiratory and Critical Care Medicine*.

[3] van Amersfoort ES, van Berkel TJC, Kuiper J (2003). Receptors, mediators, and mechanisms involved in bacterial sepsis and septic shock. *Clinical Microbiology Reviews*.

[4] Chawla-Sarkar M, Lindner DJ, Liu YF (2003). Apoptosis and interferons: role of interferon-stimulated genes as mediators of apoptosis. *Apoptosis*.

[5] Idvall J, Ahlgren I, Aronsen KF, Stenberg P (1979). Ketamine infusions: pharmacokinetics and clinical effects. *British Journal of Anaesthesia*.

[6] Beutler B, Cerami A (1988). Tumor necrosis, cachexia, shock, and inflammation: a common mediator. *Annual Review of Biochemistry*.

[7] Tartaglia LA, Goeddel DV (1992). Two TNF receptors. *Immunology Today*.

[8] Vandenabeele P, Declerq W, Beyaert R, Fiers W (1995). Two tumour necrosis factor receptors: structure and function. *Trends in Cell Biology*.

[9] Spentzas T, Kudumula R, Acuna C (2011). Role of bacterial components in macrophage activation by the LAC and MW2 strains of community-associated, methicillin-resistant *Staphylococcus aureus*. *Cellular Immunology*.

[10] English BK, Maryniw EM, Talati AJ, Meals EA (2006). Diminished macrophage inflammatory response to *Staphylococcus aureus* isolates exposed to daptomycin versus vancomycin or oxacillin. *Antimicrobial Agents and Chemotherapy*.

[11] Spentzas T, Shapley RKH, Aguirre CA (2011). Ketamine inhibits tumor necrosis factor secretion by RAW264.7 murine macrophages stimulated with antibiotic-exposed strains of community-associated, methicillin-resistant *Staphylococcus aureus*. *BMC Immunology*.

[12] Reddy SA, Huang JH, Liao WS (2000). Phosphatidylinositol 3-kinase as a mediator of TNF-induced NF-*κ*B activation. *Journal of Immunology*.

[13] Li N, Lee B, Liu RJ (2010). mTOR-dependent synapse formation underlies the rapid antidepressant effects of NMDA antagonists. *Science*.

[14] Lu YF, Tomizawa K, Moriwaki A (1996). Calcineurin inhibitors, FK506 and cyclosporin A, suppress the NMDA receptor-mediated potentials and LTP, but not depotentiation in the rat hippocampus. *Brain Research*.

[15] Sehgal SN, Baker H, Vezina C (1975). Rapamycin (AY 22,989), a new antifungal antibiotic. II. Fermentation, isolation and characterization. *Journal of Antibiotics*.

[16] Bottino R, Fernandez LA, Ricordi C (1998). Transplantation of allogeneic islets of Langerhans in the rat liver: effects of macrophage depletion on graft survival and microenvironment activation. *Diabetes*.

[17] Hartner WC, van der Werf WJ, Lodge JPA (1995). Effect of rapamycin on renal allograft survival in canine recipients treated with antilymphocyte serum, donor bone marrow, and cyclosporine. *Transplantation*.

[18] Kahan BD (2000). Efficacy of sirolimus compared with azathioprine for reduction of acute renal allograft rejection: a randomised multicentre study. *The Lancet*.

[19] Kahan BD, Julian BA, Pescovitz MD, Vanrenterghem Y, Neylan J (1999). Sirolimus reduces the incidence of acute rejection episodes despite lower cyclosporine doses in caucasian recipients of mismatched primary renal allografts: a phase II trial. *Transplantation*.

[20] Sacks SH (1999). Rapamycin on trial. *Nephrology Dialysis Transplantation*.

[21] Sigrist S, Ebel N, Langlois A (2005). Role of chemokine signaling pathways in pancreatic islet rejection during allo- and xenotransplantation. *Transplantation Proceedings*.

[22] Pritchard DI (2005). Sourcing a chemical succession for cyclosporin from parasites and human pathogens. *Drug Discovery Today*.

[23] McKeon F (1991). When worlds collide: immunosuppresants meet protein phosphatases. *Cell*.

[24] Wang T, Donahoe PK, Zervos AS (1994). Specific interaction of type I receptors of the TGF-*β* family with the immunophilin FKBP-12. *Science*.

[25] Brown EJ, Albers MW, Shin TB (1994). A mammalian protein targeted by G1-arresting rapamycin-receptor complex. *Nature*.

[26] Moore PA, Rosen CA, Carter KC (1996). Assignment of the human FKBP12-rapamycin-associated protein (FRAP) gene to chromosome 1p36 by fluorescence in situ hybridization. *Genomics*.

[27] Chung J, Kuo CJ, Crabtree GR, Blenis J (1992). Rapamycin-FKBP specifically blocks growth-dependent activation of and signaling by the 70 kd S6 protein kinases. *Cell*.

[28] Huang S, Bjornsti MA, Houghton PJ (2003). Rapamycins: mechanism of action and cellular resistance. *Cancer Biology and Therapy*.

[29] Sabers CJ, Martin MM, Brunn GJ (1995). Isolation of a protein target of the FKBP12-rapamycin complex in mammalian cells. *The Journal of Biological Chemistry*.

[30] Hay N, Sonenberg N (2004). Upstream and downstream of mTOR. *Genes and Development*.

[31] Adkins JR, Castresana MR, Wang Z, Newman WH (2004). Rapamycin inhibits release of tumor necrosis factor-*α* from human vascular smooth muscle cells. *American Surgeon*.

[32] Woltman AM, de Fijter JW, Kamerling SWA (2001). Rapamycin induces apoptosis in monocyte- and CD34-derived dendritic cells but not in monocytes and macrophages. *Blood*.

[33] Barilli A, Visigalli R, Sala R (2008). In human endothelial cells rapamycin causes mTORC2 inhibition and impairs cell viability and function. *Cardiovascular Research*.

[34] Buckingham SC, McDougal LK, Cathey LD (2004). Emergence of community-associated methicillin-resistant *Staphylococcus aureus* at a Memphis, Tennessee Children’s Hospital. *Pediatric Infectious Disease Journal*.

[35] (1999). Four pediatric deaths from community-acquired methicillin-resistant *Staphylococcus aureus*—Minnesota and North Dakota, 1997–1999. *Morbidity and Mortality Weekly Report*.

[36] Wang R, Braughton KR, Kretschmer D (2007). Identification of novel cytolytic peptides as key virulence determinants for community-associated MRSA. *Nature Medicine*.

[37] Feng N, Vollenweider FX, Minder EI, Rentsch K, Grampp T, Vonderschmitt DJ (1995). Development of a gas chromatography-mass spectrometry method for determination of ketamine in plasma and its application to human samples. *Therapeutic Drug Monitoring*.

[38] Parkin MC, Turfus SC, Smith NW (2008). Detection of ketamine and its metabolites in urine by ultra high pressure liquid chromatography-tandem mass spectrometry. *Journal of Chromatography B*.

[39] Wieber J, Gugler R, Hengstmann JH, Dengler HJ (1975). Pharmacokinetics of ketamine in man. *Anaesthesist*.

[40] Cohen J, Abraham E (1999). Microbiologic findings and correlations with serum tumor necrosis factor-*α* in patients with severe sepsis and septic shock. *Journal of Infectious Diseases*.

[41] Kawasaki C, Kawasaki T, Ogata M, Nandate K, Shigematsu A (2001). Ketamine isomers suppress superantigen-induced proinflammatory cytokine production in human whole blood. *Canadian Journal of Anesthesia*.

[42] Kawasaki T, Ogata M, Kawasaki C, Ogata JI, Inoue Y, Shigematsu A (1999). Ketamine suppresses proinflammatory cytokine production in human whole blood in vitro. *Anesthesia and Analgesia*.

[43] Koga K, Ogata M, Takenaka I, Matsumoto T, Shigematsu A (1994). Ketamine suppresses tumor necrosis factor-*α* activity and mortality in carrageenan-sensitized endotoxin shock model. *Circulatory Shock*.

[44] Girardin E, Dayer JM (1993). Cytokines and antagonists in septic shock. *Schweizerische Medizinische Wochenschrift*.

[45] Waage A, Halstensen A, Espevik T (1987). Association between tumour necrosis factor in serum and fatal outcome in patients with meningococcal disease. *The Lancet*.

[46] Offner F, Philippe J, Vogelaers D (1990). Serum tumor necrosis factor levels in patients with infectious disease and septic shock. *Journal of Laboratory and Clinical Medicine*.

[47] Calandra T, Baumgartner JD, Grau GE (1990). Prognostic values of tumor necrosis factor/cachectin, interleukin-1, interferon-*α*, and interferon-*γ* in the serum of patients with septic shock. *Journal of Infectious Diseases*.

[48] Riche F, Panis Y, Laisne MJ (1996). High tumor necrosis factor serum level is associated with increased survival in patients with abdominal septic shock: a prospective study in 59 patients. *Surgery*.

[49] Shah OJ, Wang Z, Hunter T (2004). Inappropriate activation of the TSC/Rheb/mTOR/S6K cassette induces IRS1/2 depletion, insulin resistance, and cell survival deficiencies. *Current Biology*.

[50] Manning BD, Logsdon MN, Lipovsky AI, Abbott D, Kwiatkowski DJ, Cantley LC (2005). Feedback inhibition of Akt signaling limits the growth of tumors lacking Tsc2. *Genes and Development*.

[51] Guertin DA, Sabatini DM (2005). An expanding role for mTOR in cancer. *Trends in Molecular Medicine*.

[52] Sarbassov DD, Ali SM, Sengupta S (2006). Prolonged rapamycin treatment inhibits mTORC2 assembly and Akt/PKB. *Molecular Cell*.

